# Detecting likely germline variants during tumor-based molecular profiling

**DOI:** 10.1172/JCI190264

**Published:** 2025-08-01

**Authors:** Diana Jaber, Jessica Zhang, Lucy A. Godley

**Affiliations:** 1 Department of Medicine, Northwestern Medicine, Chicago, Illinois, USA.; 2 Division of Hematology/Oncology, Department of Medicine, Robert H. Lurie Comprehensive Cancer Center, Northwestern University, Chicago, Illinois, USA.

## Abstract

As the use of molecular profiling of tumors expands, cancer diagnosis, prognosis, and treatment planning increasingly rely on the information it provides. Although primarily designed to detect somatic variants, next-generation sequencing (NGS) tumor-based profiling also identifies germline DNA alterations, necessitating careful clinical interpretation of the data. Traditionally, germline risk testing has depended on prioritizing individuals based on physical exam findings consistent with known hereditary cancer syndromes, tumor-specific features, age at diagnosis, personal history, and family history. As NGS-based molecular profiling is used increasingly to diagnose, prognosticate, and follow cancer progression, DNA variants that are likely to be of germline origin are identified with increased frequency. Because pathogenic/likely pathogenic germline variants are critical biomarkers for risk stratification and treatment planning, consensus guidelines are expanding to recommend comprehensive germline testing for more cancer patients. This Review highlights the nuances of identifying DNA variants of potential germline origin incidentally at the time of NGS-based molecular profiling and emphasizes key differences between comprehensive germline versus tumor-based platforms, sample types, and analytical methodologies. In the growing era of precision oncology, clinicians should be adept at navigating these distinctions to optimize testing strategies and leverage insights regarding germline cancer risk surveillance and management for all people with cancer.

## Introduction

Tumor-based profiling, typically performed using panels based on next-generation sequencing (NGS), is utilized widely to identify driver DNA alterations that inform diagnosis, prognosis, and targeted intervention. Although it is primarily designed to characterize the tumor somatic landscape, this approach also identifies germline variants, since all non-germ cells in the body contain inherited DNA. Germline cancer predisposition often drives tumorigenesis (1–3), underscoring the importance of germline variant detection during tumor-based profiling. Deleterious germline variants have been implicated in many cancer susceptibility genes (CSGs), several of which give rise to clinical phenotypes that inform the use of targeted therapies (4–7). As germline variants gain recognition as predictive biomarkers of therapeutic response, clinical guidelines for both solid and hematopoietic malignancies (HMs) now advocate for more comprehensive germline testing (8–21). However, tumor-based profiling alone cannot reliably distinguish between somatic and germline origin of variants without further confirmatory germline analysis. As the number of genes linked to germline cancer predisposition expands, oncology providers must be equipped to identify variants from tumor-based profiling that are of potential germline origin.

## The role of germline pathogenicity in tumorigenesis

**Figure 1:**
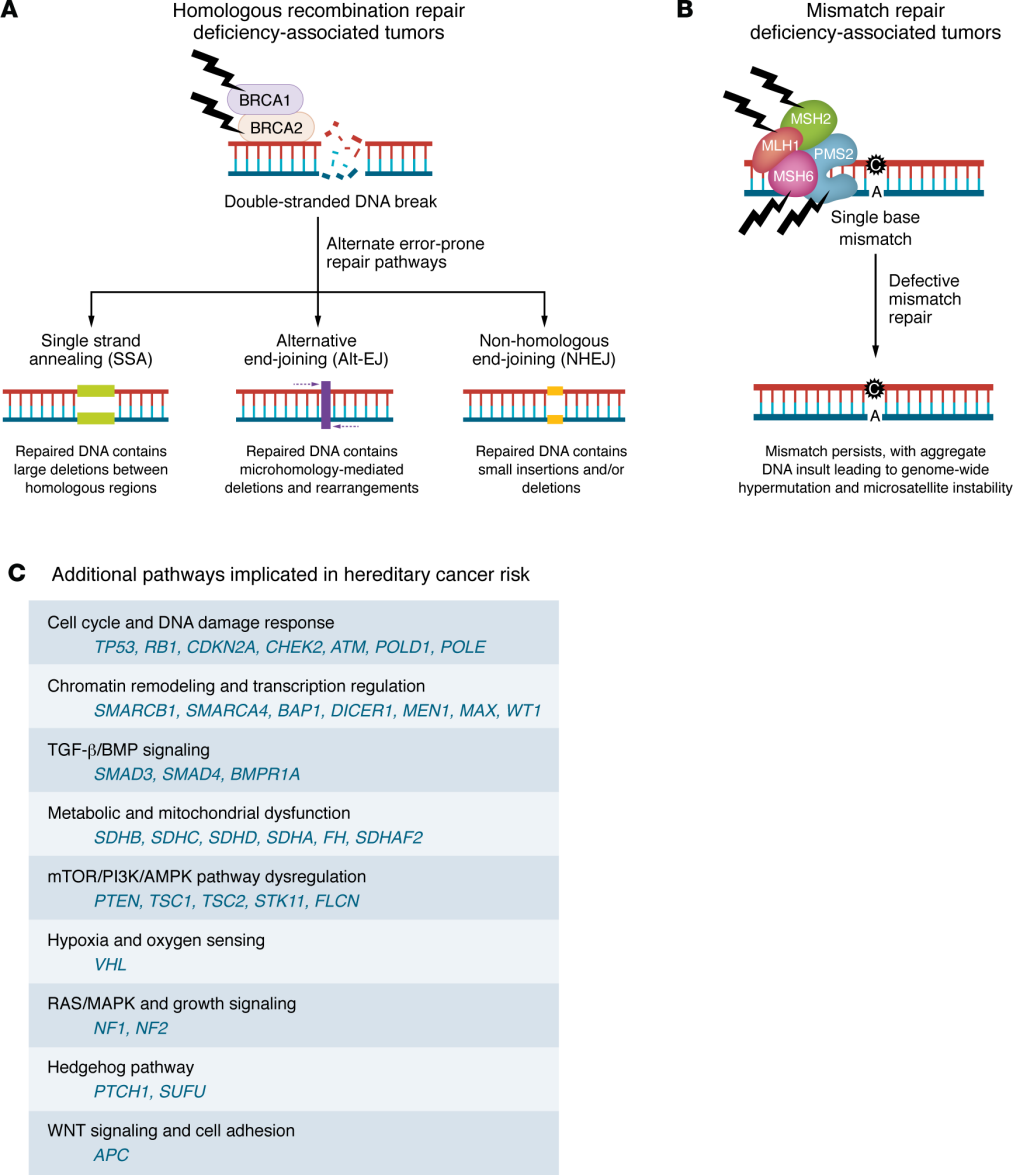
DNA repair pathways and additional mechanisms underlying hereditary cancer risk. (**A**) Defects in the homologous recombination repair (HRR) genes *BRCA1* and *BRCA2* impair the accurate repair of double-strand DNA breaks, resulting in error-prone mechanisms like single-strand annealing, alternative end joining, and non-homologous end joining, leading to increased genomic instability and the accumulation of somatic variants. HRR deficiency is associated with several tumor types, including breast, ovarian, prostate, and pancreatic cancers. (**B**) Defects in the mismatch repair (MMR) genes *MLH1*, *MSH2*, *MSH6*, and *PMS2* impair the repair of DNA replication errors, leading to microsatellite instability and genome-wide hypermutation. MMR deficiency is commonly associated with Lynch syndrome, predisposing individuals to a variety of cancers, including colorectal, endometrial, and ovarian cancers. Similar to HRR defects, MMR defects result in a mutator phenotype that drives tumorigenesis by allowing the accumulation of somatic variants. (**C**) Additional pathways implicated in hereditary cancer risk are shown, highlighting commonly altered cancer susceptibility genes recommended for further germline evaluation by the ACMG and the ESMO PMWG when identified on tumor-based profiling. Corresponding clinical phenotypes and penetrance estimates are detailed in Table 1.

Deleterious germline variants disrupt the function of CSGs that encode components integral to DNA repair, cell cycle regulation, telomere biology, and other essential cellular processes. Defects in homologous recombination repair (HRR) genes, such as *ATM*, *CHEK2*, *BRCA1*, and *BRCA2* (22), impair the accurate repair of double-strand DNA breaks. Consequently, cells rely on error-prone DNA repair mechanisms like single-strand annealing (SSA) or non-homologous end joining (NHEJ), leading to increased genomic instability and the accumulation of somatic variants (23, 24) (Figure 1). For instance, SSA promotes chromosomal rearrangements, deletions, and amplifications that drive oncogenesis, as seen in hereditary breast and ovarian cancers (25). Similarly, the upregulation of alternative NHEJ components, including LIG3 and PARP1, contributes to disease progression (26), as observed in chronic myeloid leukemia (27) and lung adenocarcinoma (28), as well as treatment resistance. In multiple myeloma, for example, *PARP1* overexpression has been implicated in resistance to melphalan, a chemotherapeutic agent (29). Moreover, in *BRCA1*-deficient tumors, the loss of LIG3 can revert resistance to poly(ADP-ribose) polymerase (PARP) inhibitors by exposing single-strand DNA gaps, highlighting alternative NHEJ as a mediator of drug resistance (30).

Beyond HRR defects, disruptions in mismatch repair (MMR) pathway genes, like *MLH1*, *MSH2*, *MSH6*, and *PMS2*, compromise DNA replication error correction, leading to microsatellite instability (MSI), a hallmark of Lynch syndrome–associated cancers, promoting genomic instability and tumorigenesis (31) (Figure 1). Germline alterations in other genes also contribute to cancer predisposition through diverse mechanisms. For instance, loss-of-function mutations in *CDH1*, which encodes E-cadherin, compromise epithelial integrity and promote invasion, predisposing individuals to hereditary diffuse gastric cancer and lobular breast cancer (32, 33). Similarly, mutations in *APC*, a regulator of the Wnt signaling pathway, result in unchecked β-catenin activation, driving the development of colorectal adenomas and carcinomas (34). These germline cancer risk alleles predispose individuals to specific cancer types and also shape the mutational landscape of tumors (Table 1).

The mode by which deleterious germline variants influence tumorigenesis varies considerably. In some cases, these alleles serve as initial driver events, whereas in others, cancer arises sporadically through independent biological pathways (2). Germline and somatic aberrations cooperate to drive cancer initiation and progression through diverse mechanisms dependent on factors such as tumor lineage and penetrance, defined as the proportion of individuals carrying a variant who develop the associated phenotype (35). Penetrance also varies significantly across genes and even within the same gene. High-penetrance variants are associated with a greater likelihood of cancer development, whereas low-penetrance variants confer a more modest risk. Srinivasan et al. identified two major routes by which germline variants influence tumorigenesis based on the analysis of pathogenic variants in 17,512 sequenced patients with cancer (2). In carriers of high-penetrance CSGs with deleterious germline variants, lineage-dependent selective pressure for biallelic inactivation in associated cancer types (e.g., *BRCA1/2* in hereditary breast cancer) demonstrated earlier age of cancer onset, fewer somatic drivers, and characteristic somatic features suggestive of dependence on the germline allele for tumor development. In this context, the germline alteration likely served as the initiating oncogenic event, with subsequent somatic events accelerating tumor formation and progression. In contrast, 27% of tumors in carriers of high-penetrance deleterious variants, and most cancers in carriers of lower-penetrance variants, did not show somatic loss of the wild-type allele or indicators of germline dependence, suggesting that the heterozygous germline variant may not have played a significant role in tumor pathogenesis. Interestingly, nearly half of the patients with high-penetrance CSGs carrying deleterious germline alleles developed cancers not typically associated with these genes, regardless of the presence of biallelic inactivation.

Although it remains unclear whether heterozygous deleterious variants foster an environment conducive to tumorigenesis, particularly in non-hereditary cancers, one potential explanation for this phenomenon is haploinsufficiency. In this scenario, a single functional allele fails to produce sufficient gene product to maintain normal cellular function, leading to abnormal phenotypes (36). Haploinsufficiency is often implicated in dosage-sensitive genes, where proper function relies on a precise amount of gene product (36). Examples of haploinsufficient genes implicated in solid and hematopoietic malignancies include those encoding transcription factors, such as CUX1 (37, 38) and Nkx3.1 (39), and tumor suppressors like p53 (40) and CHD5 (41). The degree to which haploinsufficiency contributes to disease in individuals with deleterious germline variants, particularly in cancers exhibiting incomplete penetrance, remains an area of active investigation (42–44).

Importantly, not all germline variants identified are deleterious. Germline pathogenicity is assessed through the integrated evaluation of population frequency, disease phenotype, functional data, familial segregation patterns, and predictive modeling, which together inform clinical significance. Variants are classified using the American College of Medical Genetics and Genomics (ACMG)/Association for Molecular Pathology (AMP) five-tier system as pathogenic (P), likely pathogenic (LP), variant of uncertain significance (VUS), likely benign, or benign (45). Pathogenic variants demonstrate strong evidence of disease association, such as cosegregation with cancer in families, functional studies showing detrimental effects, or a high prevalence in affected individuals. Likely pathogenic variants lack the full spectrum of evidence required for pathogenicity but still exhibit strong indications of disease association. Both P and LP variants are clinically actionable.

To standardize clinical reporting, organizations such as the ACMG and the European Society for Medical Oncology Precision Medicine Working Group (ESMO PMWG) highlight specific CSGs for additional evaluation during tumor-based profiling (46–48). For example, the ACMG recommends reporting findings from at least 28 CSGs as secondary or incidental findings (46), and the ESMO PMWG updated its guidelines in 2022 to include 40 CSGs based on data from over 49,000 tumor-normal paired samples (47, 48). These genes were selected based on their high germline conversion rate (>5% proportion that are of true germline origin), pathogenicity classification (P/LP), and high penetrance, warranting further clinical attention (Table 1).

As our understanding of germline pathogenicity evolves, so too does the process of variant classification. ClinVar (ncbi.nlm.nih.gov/clinvar) is an open-source platform that serves as a centralized repository for variant data (49, 50), where clinical laboratories and others deposit DNA variants identified in individuals along with their classifications. Clinical Genome Resource (ClinGen; https://clinicalgenome.org/) comprises expert panels who develop gene curation rules and then apply those rules to classify variants deposited into ClinVar, providing consistency in variant curation across the world. These curation panels review variants every two years to integrate new scientific literature and update variant classifications accordingly.

## Incidental germline variants: how common are they?

Numerous large pan-cancer studies have examined the frequency of incidental germline variant detection in patients who have undergone tumor-based sequencing, reporting a prevalence of 3%–17% across extensive cohort analyses (51–64) (Table 2). The majority of these studies used paired tumor-normal sequencing, in which tumor-based samples are compared with non-malignant tissue from the same patient to determine the true rate of P/LP germline variants. The wide range of reported P/LP germline variants seen across studies is likely due to multiple factors, including differences in study populations, sequencing techniques, and the number of CSGs evaluated. Earlier studies cite frequencies closer to 3%–4% (59–61), but advances in sequencing techniques in addition to larger cohort sizes likely explain the consistently higher prevalence rates observed in more recent analyses. In the largest pan-cancer study of its kind, Tung et al. examined comprehensive genomic profiling data in over 125,000 patients with advanced cancer across a wide range of solid and hematopoietic malignancies and found that 9.7% of patients harbored P/LP germline variants (51). Notably, P/LP germline variants were inferred based on a list of CSGs, ClinVar evidence, and variant allele frequency thresholds, rather than confirmed via paired tumor-normal sequencing. Another notable large pan-cancer study that used paired tumor-normal sequencing found that among 10,389 individuals across 33 cancer types, 8% of patients carried P/LP germline variants with considerable variability by cancer type, ranging in prevalence from 22.9% in pheochromocytoma/paraganglioma and 19.9% in ovarian cancer to 2.2% in cholangiocarcinoma (56). In another recent study, Stadler et al. found the prevalence of P/LP germline variants to be as high as 17%, which may reflect differences in cohort composition, as their study included younger patients (median age at diagnosis, 58 years) and a higher proportion with more advanced disease (81% stage IV malignancies) (53). Taken together, the true frequency of incidental germline variants detected in patients who have undergone tumor-based profiling likely approximates 10%, representing a substantial proportion of patients.

Notably, Tung et al. found P/LP germline variants in similar proportions between cancers with formal National Comprehensive Cancer Network (NCCN) recommendations for universal testing (e.g., epithelial ovarian cancer, metastatic prostate cancer, pancreatic adenocarcinoma) and all other cancer types (11% and 9%, respectively) (51). The most frequently identified genes with potential P/LP germline variants included *BRCA2* (16.9%), *MUTYH* (15.0%), *ATM* (13.4%), *CHEK2* (11.7%), and *BRCA1* (9.8%). Importantly, the majority (64%) of germline variants were discovered in patients who would not have been recommended for germline testing based on current guidelines. For example, P/LP germline variants were detected in over 2,000 (7.1%) patients with lung cancer, a group for whom universal testing is not currently recommended. Similar findings were reported in a study by Yap et al., which found that among 9,279 patients with six cancer types notably lacking hereditary cancer guidelines (e.g., bladder, brain, lung, bile duct, esophageal, and head/neck cancers), 6.5% harbored incidental P/LP germline variants (52).

In addition to pan-tumor studies, others have investigated the prevalence of incidental P/LP germline variants detected in certain subpopulations. Drazer et al. reviewed NGS panels in 360 patients with HMs, focusing on deleterious variants in nine genes associated with hereditary HMs, including *DDX41*, *GATA2*, *RUNX1*, and *TP53*. Among 52 P/LP variants detected in 44 patients, 12% (6/52) were confirmed as germline through paired tumor–normal analyses of germline tissue (57). In studies investigating the prevalence of germline P/LP variants in pediatric populations with cancer, 8%–12% of pediatric patients were found to harbor such genetic variants (62–64). In one such study by Zhang et al., the CSGs most commonly implicated included *TP53*, *APC*, *BRCA2*, *NF1*, *PMS2*, *RB1*, and *RUNX1* (63). Notably, the study found that only 40% of patients with P/LP germline mutations had a positive family history of cancer, comparable to 42% of randomly selected pediatric patients without germline variants, suggesting that family history alone is not a reliable predictor of underlying germline predisposition in pediatric cancer patients. Together, these findings underscore the prevalence of suspected deleterious germline variants detected during tumor-based profiling and support the notion that P/LP germline variants are far more common than has been traditionally thought. Such findings further suggest that expanded germline testing may help to capture at-risk patients and their relatives who would not have met existing screening criteria, with the potential to profoundly inform future genetic counseling, risk stratification, and targeted therapeutics. Moreover, given that tumor-only sequencing has been shown to miss a significant number of germline variants (65, 66) (as discussed below in “Germline versus tumor-based profiling in cancer: current approaches”), the adoption of comprehensive germline testing may reveal an even greater frequency of P/LP variants, particularly in cancers not historically associated with inherited risk.

Because comprehensive germline analysis is central to uncovering novel predisposition genes and identifying new biological pathways, such as epigenetic regulation and tyrosine kinase signaling in heritable oncogenesis (63, 67–69), broader testing could substantially expand our understanding of cancer etiology. These findings have led to a growing reconsideration of what constitutes “hereditary” versus “sporadic” cancer, suggesting that many cases previously labeled as sporadic may, in fact, arise on a continuum of germline susceptibility (2, 70, 71). As such, the broader adoption of germline sequencing not only offers tangible benefits for treatment and prevention but also has the potential to reshape foundational concepts of cancer origin, risk stratification, and classification (2, 56, 70, 71).

## Implications of germline variant detection: clinical applications

### Targeted therapeutic management.

Proper distinction between somatic and germline variants has important clinical implications for patients in both early- and late-stage cancers. From a therapeutic perspective, the emergence of immune checkpoint inhibitors (ICIs) like pembrolizumab and nivolumab has revolutionized the treatment of microsatellite instability–high (MSI-H) or defective mismatch repair (dMMR) cancers, commonly linked to germline variants in MMR genes (e.g., *MLH1*, *MSH2*, *MSH6*, *PMS2*) (72, 73). By blocking the PD-1/PD-L1 pathway, ICIs demonstrate durable therapeutic responses in colorectal (74), endometrial (75), non-colorectal digestive (76), and other Lynch syndrome cancers with MSI-H/dMMR phenotypes. Similarly, P/LP germline *BRCA1/2* variants confer heightened sensitivity to PARP inhibitors like olaparib, which is FDA-approved in *BRCA*-mutated breast (77, 78), ovarian (79, 80), pancreatic (81), and prostate cancers (82, 83), in both the early and metastatic settings. Moreover, patients harboring germline HRR alterations, including *ATM*, *CHEK2*, *BRCA1/2*, *PALB2*, *RAD51D*, and *BAP1*, are particularly susceptible to the use of platinum-based chemotherapies, which exploit DNA repair deficiencies conferred by HRR dysfunction (84–87).

Importantly, the efficacy of targeted therapies relies on the deleterious germline variant being the primary “driver” of tumorigenesis. For example, although PARP inhibitors represent a revolutionary advancement in precision medicine, their efficacy in people with germline *BRCA1/2* cancer risk alleles with non-*BRCA*-associated cancer types may be limited (88). Jonsson et al. found that among advanced-cancer patients with deleterious germline *BRCA1/2* alleles (2.7%) or somatic loss-of-function alterations (1.8%), selective pressure for biallelic gene inactivation and PARP inhibitor sensitivity was only observed in *BRCA*-associated cancers (88). Conversely, non-*BRCA*-associated tumors appeared to develop and evolve independently of the *BRCA1/2* germline variant (88). Clinicians should recognize that the presence of a deleterious germline variant in a CSG with traditionally high penetrance does not necessarily portend therapeutic responsiveness, particularly in cancers not associated with the disorder (2).

### Surveillance and risk reduction strategies.

Individuals with inherited cancer predisposition benefit from variant-specific surveillance and management strategies. For example, it is recommended that germline *TP53* carriers undergo annual whole-body and brain MRIs, along with enhanced colorectal cancer screening every 2–5 years, to identify malignancies at localized stages, allowing for potentially curative treatment (89). Further, modifications in radiation therapy protocols may be necessary to reduce the risk of secondary cancers (90). Asymptomatic carriers of germline *ERCC6L2* variants should undergo regular hematologic surveillance, including bone marrow testing, cytogenetic analysis, and tumor-based profiling, with a focus on detecting somatic *TP53* variants, as these may progress to acute myeloid leukemia (AML) (91). Moreover, detection of somatic *TP53* variants in HMs portends inferior responses to conventional chemotherapy. Therapies specifically targeted against acquired variants, such as vemurafenib against *BRAF* V600E in hairy cell leukemia (92), now exist.

In the case of HMs, identification of deleterious germline variants is critical for donor consideration in allogeneic hematopoietic stem cell transplantation (allo-HSCT), as family members are generally preferred (93). Deleterious variants in genes such as *CEBPA*, *DDX41*, *GATA2*, and *RUNX1* have been associated with inferior allo-HSCT outcomes (94–97), including donor-derived malignancies (94–97), failure or delay in engraftment (98, 99), severe graft-versus-host disease (100), impaired immune reconstitution (101), and early relapse (98). Currently, there is no standardized approach for mandated donor germline testing in allo-HSCT, leaving the possibility that matched unrelated donors may also carry germline cancer risk alleles, especially those relatively common in certain populations (102).

Identifying hereditary risk can prompt cascade testing for family members, enabling targeted screening and risk-reducing interventions (103). Cascade testing begins with identification of an individual (proband) diagnosed with a hereditary cancer syndrome and confirmation of a deleterious germline cancer risk allele. At-risk relatives can then be identified, informed, and offered genetic testing. Notably, direct communication from the clinical team to relatives significantly improves the uptake of cascade testing, facilitating earlier detection and implementation of risk reduction strategies (104, 105).

### Guideline-based germline testing: challenges of limited gene coverage.

Many clinical practice guidelines advocate for more expansive germline testing. Guidelines from the American Society of Clinical Oncology (ASCO) (8, 9, 20, 21, 106) and the NCCN (15, 17, 107–109) recommend universal germline testing in patients with epithelial ovarian cancer, pancreatic adenocarcinoma, metastatic or high-risk prostate cancer, pleural mesothelioma, adrenocortical carcinoma, pheochromocytomas, or paragangliomas, regardless of age, family history, or personal history. For other malignancies, such as breast, colorectal, and endometrial cancers, guidelines support germline testing based on simplified clinical criteria such as age at diagnosis, tumor subtype, or universal MMR tumor screening to identify at-risk individuals, even in the absence of family history (15, 16). However, recommendations for universal testing across many other cancer types, regardless of clinical or familial risk, remain underdeveloped (48, 106, 110, 111). These recommendations, while evolving, leave gaps in testing for patients with cancers outside of high-priority groups, in whom underlying genetic drivers may still play a crucial role in tumorigenesis. Moreover, current guidelines emphasize multi-gene panel testing that targets a subset of high-penetrance genes, such as *BRCA1/2*, *MLH1*, and *MSH2*, associated with well-established hereditary cancer syndromes and P/LP variants with known clinical significance (15, 16, 48, 106, 110). Although effective for identifying common germline predisposition alleles, this strategy may exclude less studied variants in genes that may confer heritable risk, particularly in atypical or rare cancer syndromes. In these cases, a broader, more inclusive testing approach is necessary, but such options are not yet standard in guidelines, which dictate the scope of clinical action and management strategies. Consequently, this limitation may lead to missed diagnoses in patient populations whose genetic risk profiles do not align with currently defined criteria (51, 53).

**Figure 2:**
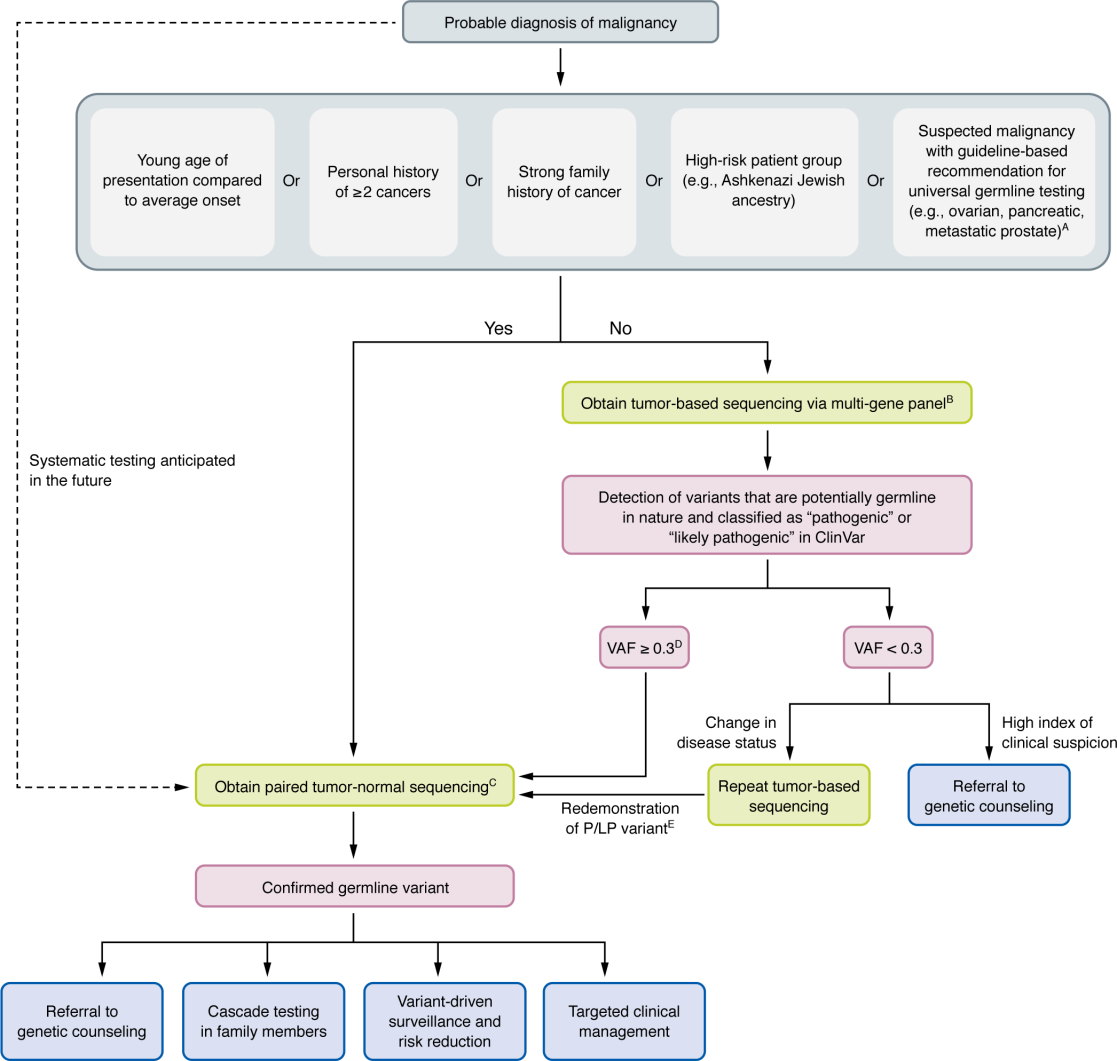
Proposed algorithm to aid the identification of likely germline variants during tumor-based profiling. In a patient with a probable malignancy (i.e., before pathologic confirmation), a thorough clinical history should be performed to determine risk. High-risk patients are prioritized for paired tumor-normal sequencing to differentiate between somatic and germline alterations. For patients not identified as high-risk who undergo tumor-based profiling, variant allele frequency (VAF) thresholds and pathogenicity classifications should guide confirmatory germline testing. In patients with a VAF less than 0.3 who experience a change in disease status, clinicians should evaluate the appropriateness of additional germline testing based on the clinical context. Confirmed germline variants should prompt targeted clinical action. Shared decision-making is essential, particularly when prognosis is limited or results are unlikely to influence management. In the future (indicated by the dashed line), we anticipate standardized integration of simultaneous germline testing and tumor-based profiling at the time of diagnosis. ^A^Guidelines from ASCO and NCCN recommend universal germline testing in patients with epithelial ovarian cancer, pancreatic adenocarcinoma, metastatic or high-risk prostate cancer, pleural mesothelioma, adrenocortical carcinoma, pheochromocytomas, or paragangliomas, regardless of age, family history, or personal history (15, 17, 107–109). ^B^Gene selection for multi-gene panels should adhere to ASCO guidelines, which consider clinical relevance, actionability, penetrance, and associations with hereditary cancer syndromes (106). ^C^For paired tumor-normal testing, best practice entails using cultured skin for germline analysis. ^D^This VAF threshold is derived from ESMO PMWG, which designated a cutoff of ≥0.3 for single-nucleotide variants and ≥0.2 for indels (48). Notably, VAF alone, particularly when derived from a tumor sample at a single time point, remains insufficient to determine germline origin. ^E^VAF may fluctuate across time points because of technical variation, tumor heterogeneity, or biological phenomena such as allelic loss or subclonal architecture. Thus, redemonstration of the same P/LP variant on repeat tumor sequencing, independent of VAF, warrants consideration for germline evaluation (66, 106).

Given that the prevalence of P/LP germline variants in cancer patients likely approaches 10% at a minimum, we anticipate that recommendations for all cancer patients to undergo comprehensive germline testing will emerge soon. Without recognition of the germline cause of many cancers, there are detrimental consequences of failing to diagnose these conditions for both patients and their families. Given the significant overlap in cancer risk alleles between solid and hematopoietic malignancies, we envision a future in which tumor-agnostic cancer risk testing is recommended for all cancer patients, with increasing ability to identify these alleles from tumor-based NGS assays. Owing to financial and practical limitations, this approach may not be feasible, as accessibility to laboratories with advanced variant detection and curation programs remains constrained in the current landscape of genetic testing (112). Nonetheless, clinicians should advocate for germline testing in patients in whom they suspect germline predisposition or who meet high-risk criteria (outlined below in “Distinguishing germline variants in tumor-based profiling”) (Figure 2).

## Germline versus tumor-based profiling in cancer: current approaches

Tumor-based profiling, while informative, has limitations in detecting P/LP germline variants. In a cohort of patients with various cancer types, Terraf et al. found that tumor-only sequencing missed 10.5% of clinically actionable deleterious germline variants, particularly in homologous recombination deficiency (HRD) and MMR genes (65). Similarly, Lincoln et al. reported that 8.1% of deleterious germline variants were missed using tumor-only testing (66). These studies highlight the need for separate, comprehensive germline testing, as tumor-based panels cannot serve as an adequate substitute owing to various factors (65, 66). First, the optimal sample type for each test is distinct. Tumor-based profiling requires malignant cells, whereas germline testing relies ideally on non-malignant cells to avoid confusion with somatic variants (113). In solid tumors, buccal swabs are generally reliable for germline testing, as they provide DNA free from tumor contamination (114). However, in HMs, best practice entails using cultured skin fibroblasts for germline testing (115–117), as hematopoietic tissue (e.g., saliva, peripheral blood) is contaminated with malignant cells and can be confounded by processes like clonal hematopoiesis, and therefore somatic alterations (113, 116, 118). Clonal hematopoiesis, a form of somatic mosaicism, refers to the expansion of hematopoietic stem cell clones, which increases with age (118). As peripheral blood contains all stem cell progeny, deep sequencing can reveal acquired mutations over time that may confound germline analysis (118). Differentiating between somatic and true germline variants in the setting of mosaicism remains a challenge. For instance, somatic alterations in *TP53*, referred to as “clonal *TP53*,” can result from aberrant clonal expansion rather than true constitutional mosaicism or germline predisposition, requiring careful analysis (119–121). Somatic reversion, as seen with *SAMD9*/*SAMD9L*, occurs when a deleterious germline variant is spontaneously corrected in somatic cells, eliminating the original germline allele and further complicating interpretation (122–124). Although hair follicles and nail clippings can serve as alternative germline sources and may yield reliable results, both are limited by low DNA yield and technical constraints (125–127). Hair follicles exhibit variable DNA degradation and poor genotyping performance (128), while nail clippings are prone to tumor contamination, particularly in myeloid neoplasms (129). Given the prolonged timeline for fibroblast culture, for cases in which rapid results are required for clinical assessment, we recommend buccal swabs as the preferred initial source for germline testing in HMs (125), followed by confirmatory analysis using fibroblast-derived DNA.

Generally, although tumor-based profiling is primarily focused on identifying variants with known clinical implications, comprehensive germline platforms must remain adaptable to accommodate newly identified cancer risk genes. Thus, tumor-based and germline assays require distinct platforms. Targeted panels in tumor profiling focus on exonic regions of genes relevant to oncogenesis and therapeutic targets, given that clinically relevant acquired mutations typically occur in these regions. Because of the inherent heterogeneity of tumor samples, including varying ratios of malignant to non-malignant cells, tumor-based platforms require average coverage depths of at least 1,000-fold to detect low-frequency somatic variants, especially in tissue samples with low tumor cellularity (130). These tests are designed to detect single-nucleotide variants (SNVs) and large copy number variants (CNVs), which have therapeutic and prognostic relevance (65, 131, 132).

In contrast, germline testing offers broader coverage, including both exonic and non-coding regions (e.g., promoters and enhancers), which allows for the identification of many variant types, including small CNVs, that may be missed in tumor-based profiling (65, 131, 132). Comprehensive germline testing can be conducted through augmented whole-exome sequencing (aWES), where primers that capture selected non-coding regions supplement exonic analysis for CNV identification (65, 131, 132). Because approximately 85% of P/LP variants exist within exons (133), aWES is typically favored over whole-genome sequencing given its currently lower costs, but this may change as sequencing costs decline rapidly.

If aWES is not employed, germline multi-gene (multiplex) tests can be used, especially to query multiple high-penetrance genes or particular hereditary cancer syndromes (134). These panels involve disease-targeted exon-capture methods focused on selected genes of interest, offering higher coverage and depth for these genes when compared with aWES (135–137). In contrast to the high coverage depths in tumor-based assays, germline testing requires a minimum depth of 30-fold, as germline variants exist in heterozygous or homozygous states (138, 139). This enhanced resolution improves the detection of variants, including larger deletions or duplications, which may be missed by other methods. For example, multiplex ligation-dependent probe amplification is often used to identify deleterious structural variants in *MLH1* and *MSH2* for Lynch syndrome, or *APC* for familial adenomatous polyposis (140). By prioritizing predefined, clinically actionable genes, multi-gene panels provide a cost-effective and efficient approach for identifying hereditary predispositions. However, as noted above, they may miss cancer-associated variants in genes not included in the panel, which can be problematic in rare hereditary syndromes or atypical presentations (106).

## Distinguishing germline variants in tumor-based profiling

### Molecular and bioinformatic considerations.

The clinical classifications of variants detected by tumor profiling tests are based on their somatic nature. Given that DNA changes are often context dependent, germline alleles may have different effects compared with somatic alleles that are only present in a tumor (141, 142). For this reason, germline and somatic curations are distinct (141, 143). Clinicians should recognize variant alleles that are overwhelmingly likely to be germline to facilitate timely germline testing and confirmation. For instance, *DDX41* variants identified in AML/myelodysplastic syndrome (MDS) panels at a variant allele frequency (VAF) greater than 40% are germline in 94% of patients (144). Simon et al. found that a notable proportion (27%) of *RUNX1* variants in patients with *RUNX1*-mutant AML were of germline origin (145), and among those, 16% are deleterious when curated using *RUNX1*-specific curation rules (116, 146–148). This example underscores that germline status alone does not confer pathogenicity and highlights the importance of careful, variant-specific interpretation to guide appropriate management (116).

The VAF quantifies the proportion of specific variant alleles within a given NGS sample. For germline variants, the VAF ideally approximates 50% for heterozygous variants and 100% for homozygous variants (149). However, underestimations in germline VAF may occur for several reasons, including germline mosaicism, wherein post-zygotic mutations during early embryonic development cause the variant to be present in only a subset of cells, as well as genomic loss of the wild-type allele, CNVs in tumor cells, structural rearrangements, and technical factors such as sequencing artifacts (102, 150, 151). Although VAF alone, particularly when derived from a tumor sample at one single point in time, is not sufficient to determine germline origin, some identification methods use VAF thresholds to inform bioinformatic algorithms in distinguishing germline variants. Kraft et al. analyzed VAFs across sequential tumor samples and observed that VAFs between 0.3 and 0.7, when stable over time, were more likely to represent germline variants (152). Using coefficients of variation to assess VAF changes, variants were graded on a scale from 1 to 5, with grade 1 being the most likely to be germline and grade 5 the least likely. To validate these predictions, they performed aWES on cultured skin fibroblast DNA, confirming 89% (48 of 54) of grade 1 variants as germline. Alternative bioinformatic approaches used to flag potential germline variants compare tumor samples to a “virtual normal,” which is a composite of sequencing data from unrelated healthy individuals, used in place of the patient’s own matched normal sample (153). This approach assumes that most variants are prevalent in the general population, effectively removing over 96% of germline variants in tumor samples, but is otherwise limited in its ability to detect rare pathogenic variants (153). Jalloul et al. introduced a computational method that integrates VAF, tumor purity, and copy number to infer germline status from tumor-only sequencing, achieving 86% accuracy and a 3% false omission rate (154). Unlike threshold-based or population-filtering approaches, this gene-agnostic model provides systematic, variant-level inference without requiring matched normal tissue. Complementing these bioinformatic methods, integration of clinical genetics into tumor sequencing workflows has been shown to enhance germline variant detection; in one study, incorporation of clinical genetics during molecular tumor board review of tumor-only NGS increased detection from 1.4% to 7.5%, even among patients who did not meet standard testing criteria (155).

Other strategies utilize VAF thresholds as part of a multistep process to screen pathogenic germline variants. To aid clinicians in identifying potential germline variants in high-risk patients during tumor-based profiling, we have outlined an approach that integrates VAF thresholds, ClinVar pathogenicity classifications, and a list of CSGs with reported high rates of germline conversion, guided by ACMG and ESMO PMWG recommendations (46–48) (Figure 2).

### Paired tumor-normal analysis.

**Figure 3:**
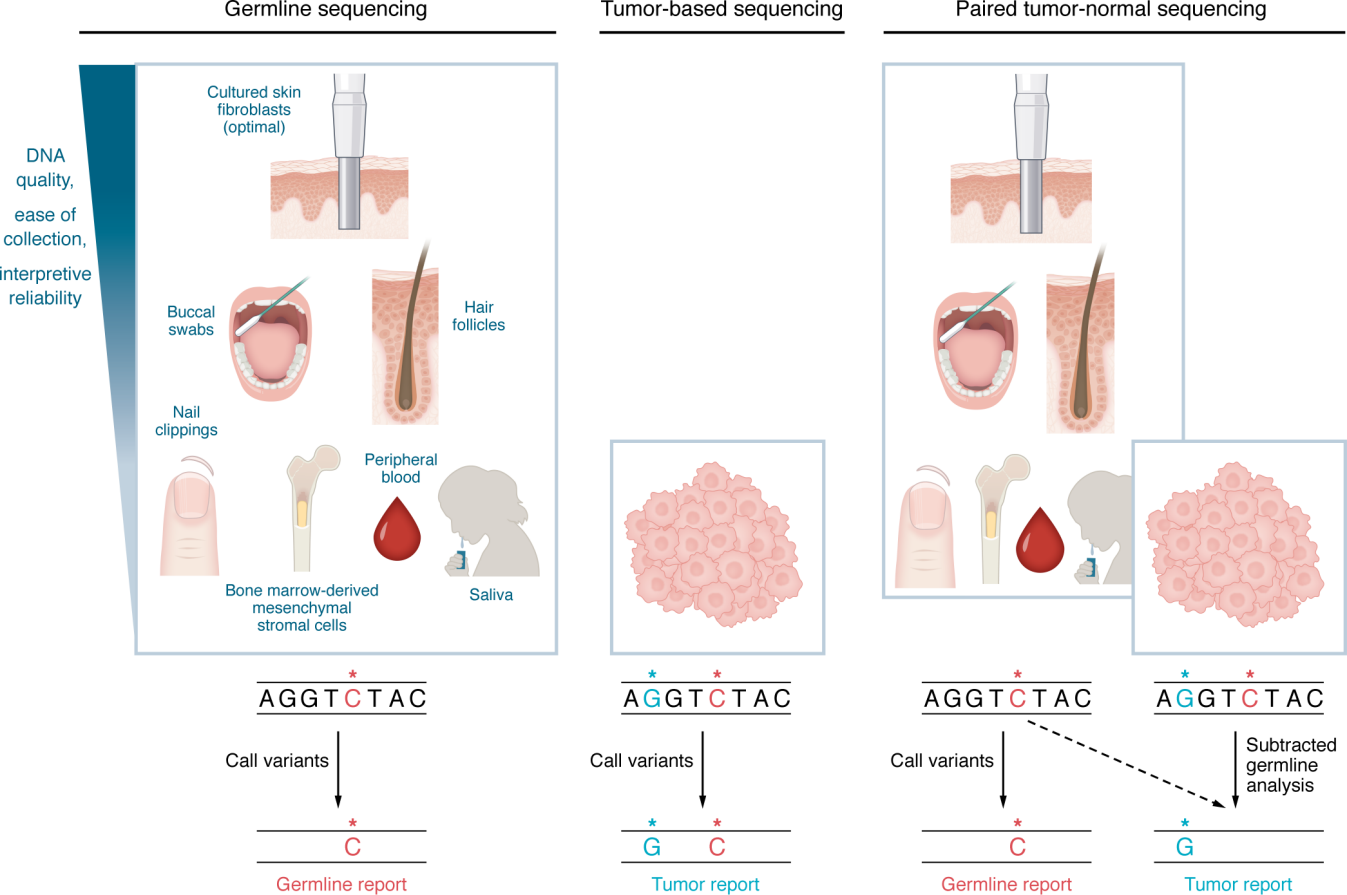
Comparison of germline, tumor-based, and paired tumor-normal sequencing. Paired tumor-normal sequencing, the gold standard for confirming incidental germline findings, involves simultaneous sequencing of tumor and matched normal, non-malignant tissue from the same patient. This approach identifies inherited variants to generate a germline report. Tumor-based profiling analyzes DNA from tumor cells, reporting both somatic alterations and potential germline variants. Germline sequencing should be performed using non-hematopoietic tissue, with cultured skin fibroblasts considered the gold standard. Alternative sources are depicted in descending order based on DNA quality, ease of collection, and interpretive reliability. By subtracting germline variants from the tumor sequence, paired testing differentiates between somatic and germline variants, improving specificity and reducing false-positive somatic calls. Unlike tumor-based profiling, paired tumor-normal sequencing provides direct discrimination between somatic and germline variants.

Paired tumor-normal testing is the most common method for confirmation of incidental germline variants detected during tumor-based profiling (65, 156, 157) (Figure 3). This approach involves the simultaneous sequencing of DNA isolated from both tumor tissue and matched normal, or non-malignant, tissue (e.g., cultured skin fibroblasts) in the same patient (157). By comparing the two sequences, paired testing differentiates inherited from acquired variants, thereby identifying heritable cancer risk even in the absence of clear family history while minimizing false-positive somatic variant detection and improving result specificity (61, 158). Although detection of a suspected germline variant through tumor-only sequencing triggers additional confirmatory testing, tumor-normal testing allows for direct discrimination between somatic and germline alterations, thereby reducing test burden (157). Although considered the gold standard, tumor-normal testing is limited by cost and logistical challenges, due in part to the requirement for an additional normal sample (159, 160).

### Patient history: clues for testing.

Specific indicators in a patient’s history that should raise suspicion for heritable cancer risk include young age of onset, personal history of two or more cancers, early-onset cancers in multiple relatives, and clinical scenarios that raise concern regarding hereditary cancer syndromes (16, 106). Generally, germline testing should be considered in an individual diagnosed with cancer prior to age 50 years within two generations of the proband (15, 16, 18, 19). Certain populations, such as individuals of Ashkenazi Jewish (AJ) ancestry, have a higher prevalence of founder mutations and should be tested if they have a personal or family history of breast, pancreatic, prostate, ovarian, or uterine cancers (15, 106). Additionally, individuals of AJ ancestry have an increased prevalence of colorectal cancer and renal cancer due to the increased frequency of the *APC* I1307K polymorphism in this population (161). Germline testing is also recommended for individuals with a first-degree relative with features suggestive of heritable cancer risk, such as early-onset or triple-negative breast cancer, male breast cancer, ovarian cancer, pancreatic cancer, or high-risk prostate cancer, even in the absence of a personal cancer diagnosis (15). Further, tumors with low prevalence in the general population, such as retinoblastoma, pheochromocytoma, or paraganglioma, should prompt evaluation for inherited cancer syndromes (109). It is also important to consider the clinical scenario within which a gene/tumor combination presents itself. For instance, a *RET* variant found in a sporadic medullary thyroid carcinoma (MTC) in a 75-year-old individual without a family history of endocrine neoplasias is unlikely to be hereditary. In contrast, the same variant in a 25-year-old with MTC and a history of pheochromocytoma may strongly suggest a germline *RET* variant associated with multiple endocrine neoplasia type 2A (MEN2A). A personal HM history, particularly when coupled with a family history of another HM, prolonged cytopenias, or other hematopoietic abnormalities, should also raise suspicion of a hereditary predisposition (18, 19, 113). In addition, the presence of hypocellular MDS or a new diagnosis of aplastic anemia should prompt further consideration for hereditary myeloid predisposition syndromes (19). Identification of a DNA variant in at least two related individuals defines its germline status, and this approach is occasionally more feasible, particularly in HMs when skin biopsy or fibroblast culture may be difficult to obtain (113).

## Reporting and disclosure of incidental germline variants

Both laboratories and ordering clinicians share the responsibility for identifying and managing incidental germline findings. Laboratories conducting tumor-based profiling must implement robust data filtering algorithms to differentiate between germline and somatic variants, conduct downstream testing to confirm potential germline variants, and establish standardized protocols for reporting findings (162). When a potential germline variant is detected, reporting should follow the standards set forth by the joint consensus guidelines by ASCO, AMP, and the College of American Pathologists (149). These guidelines emphasize reporting incidental germline variants that have known clinical impact, including those with therapeutic implications or links to hereditary cancer syndromes or that inform clinical management and surveillance strategies (149) (Table 1). Variant pathogenicity should be assessed using established criteria and determined independently of the interpretation of the cause of disease in a given patient. Variants of uncertain significance (VUS) should also be reported, but should not be used in clinical decision-making (149). Following confirmation of germline status, clinicians are responsible for determining whether identified variants, particularly those classified as P/LP, are clinically actionable within the context of the patient’s presentation. If appropriate, further actions, such as genetic counseling, cascade testing, or targeted management, should be pursued (Figure 2).

Clinicians should obtain appropriate consent through engagement of patients in a discussion of the potential for incidental germline variant detection during tumor-based profiling, explicitly addressing the implications such findings may have for medical management, familial risk assessment, and future clinical decisions. This conversation should also clarify the scope of testing, the limitations of current knowledge, and the possibility that information may evolve with advances in NGS testing.

## Conclusions

The genetic architecture of inherited cancer predisposition is complex, shaped by substantial heterogeneity in penetrance, lineage-dependence, and germline-somatic variant interactions that collectively influence cancer susceptibility. As our understanding of cancer predisposition continues to evolve, the role of germline variant detection in guiding targeted management becomes increasingly critical. The cumulative prevalence of incidental P/LP germline variant detection during tumor-based profiling approximates 10%, representing a substantial proportion of patients. Notably, detection occurs at comparable rates among cancers with and without formal guideline recommendations for germline testing. Although tumor-based profiling provides a valuable opportunity to identify clinically actionable germline variants, it does not serve as an appropriate substitute for comprehensive germline testing. Given the potentially detrimental consequences of missed variant detection for both patients and their families, we anticipate a future in which tumor-agnostic germline testing is accessible to all patients with solid and/or hematopoietic malignancies. Until such testing becomes standard, clinicians should advocate for germline testing in patients in whom they suspect germline predisposition or those who meet high-risk criteria. Moreover, with the differential effects that germline variants can exhibit on cancer pathogenesis and progression, clinicians must be equipped with the knowledge to interpret results of comprehensive genetic testing, determine clinical actionability, and appropriately counsel patients. Until the influence of germline predisposition in non-associated cancers is better understood, the emphasis on cancer screenings, genetic counseling, and disease-specific preventative strategies in patients with variants in cancer susceptibility genes should remain a priority.

## Figures and Tables

**Table 2:**
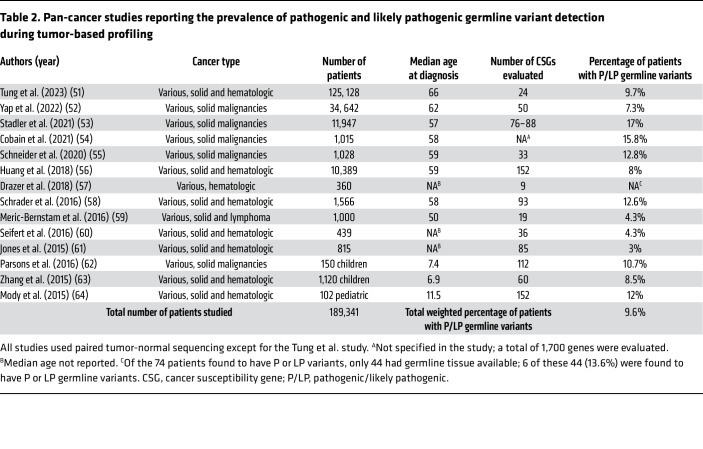
Pan-cancer studies reporting the prevalence of pathogenic and likely pathogenic germline variant detection during tumor-based profiling

**Table 1:**
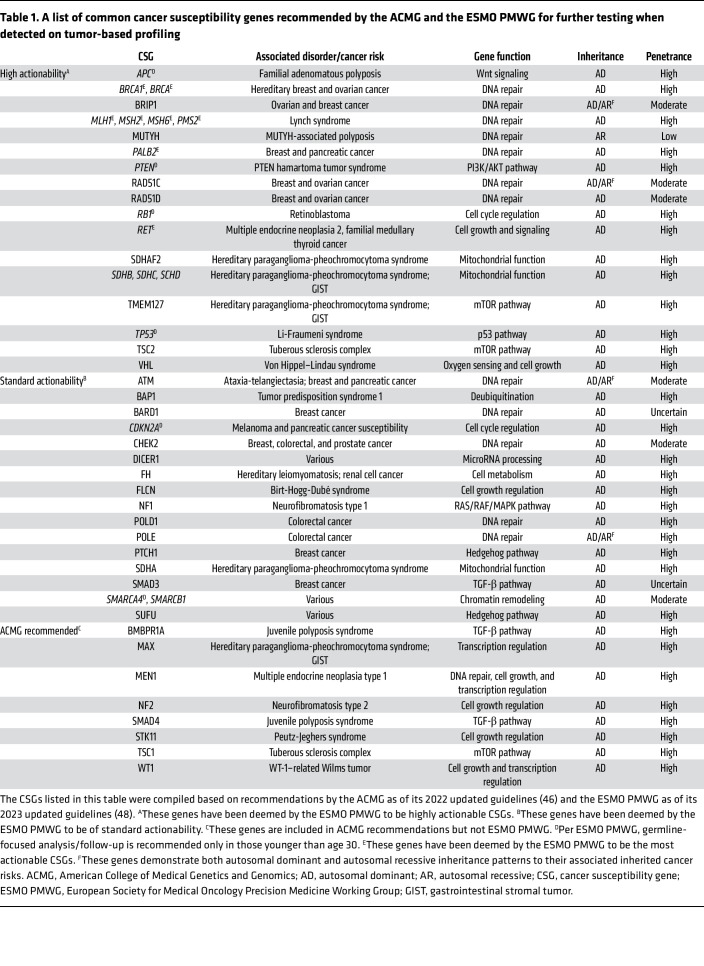
A list of common cancer susceptibility genes recommended by the ACMG and the ESMO PMWG for further testing when detected on tumor-based profiling

## Version 1 08/01/2025

Electronic publication
